# Burnout among surgeons; depression, anxiety and stress between consultant versus post-graduate trainee

**DOI:** 10.12669/pjms.36.7.1415

**Published:** 2020

**Authors:** Saleh Khurshied, Aliya Hisam, Nawal Khurshid, Madiha Khurshid

**Affiliations:** 1 Saleh Khurshied, MBBS. House officer, Pakistan Institute of Medical Sciences, Islamabad, Pakistan; 2 Dr. Aliya Hisam, MBBS, MPH, FCPS (Community Medicine). Associate Professor, Department of Community Medicine, Army Medical College, National University of Medical Sciences, Rawalpindi, Pakistan; 3 Dr. Nawal Khurshid, MBBS. Resident Otorhinolaryngologist, Pakistan Institute of Medical Sciences, Islamabad, Pakistan; 4 Dr. Madiha Khurshid, BDS, RDS, Dental Surgeon, Dental Surgeon, Al-Khidmat Razi Hospital, Islamabad, Pakistan

**Keywords:** Anxiety, Depression, Postgraduate trainees, Surgeons, Stress

## Abstract

**Objective:**

To compare depression, anxiety and stress between consultant and post-graduate trainee (PG-trainee) surgeons and to find the difference of different factors i.e. gender, marital status, physical activity, BMI, comorbidity and Income per month between the two.

**Methods:**

A descriptive cross-sectional study of eight months duration from March 2018 to August 2018 was conducted in Military Hospital and Combined Military Hospital, Rawalpindi, Pakistan. Convenient sampling technique was used. DASS 21 questionnaire was used for data collection. Data were entered and analysed by SPSS 22. A p-value of < 0.05 was considered statistically significant.

**Results:**

The mean age of participants was 37.44±10.512 years. Out of 68 participants, 54 (79.4%) were males and 14 (20.6%) were females. There was a significant difference between the consultants and PG trainees in terms of gender, marital status and income per month (p= <0.005) PG-trainees were more depressed and anxious but not stressed as compared to consultants (p= 0.014, 0.012 and 0.280 respectively).

**Conclusions:**

There was a significant association in terms of gender, marital status and income per month between consultants and PG trainees. A statistically significant association was found between consultants and PG trainees concerning depression and anxiety showing PG trainees were more depressed and anxious.

## INTRODUCTION

Stress may be a beneficial or harmful psychological state which is evoked by any intrinsic or extrinsic stimuli.^1^ Depression is a disorder of mood which affects person’s thoughts and feelings and causes a persistent feeling of sadness and loss of interest.^2^ Anxiety is an emotion identified by often with a person showing nervous behaviour, such as pacing back and forth, somatic complaints and rumination.^3^ Burnout can occur from prolonged stressors of job and it is a psychological syndrome.^4^

Burnout among surgeons is related to increase in frequency of medical errors and this can have harmful effects on patients compromising their safety and the quality of care provided.^5^ Body Mass Index (BMI), physical activity, income per month, marital status and comorbidities are the basic factors which generally affect a person both physically and psychologically and surgeons are no exception to this fact.^6^

The study aimed to compare depression, anxiety and stress between consultant and PG trainee surgeons. BMI, physical activity, marital status, income per month and comorbidities are the factors looked upon for their relation with depression, anxiety and stress. In this research, these factors are directly compared between consultant and PG trainee surgeons in our setup. This research will help know the depression, anxiety and stress levels in consultants and PG trainees and also comparing the effect of different factors.

## METHODS

This was a descriptive cross-sectional study carried out on surgeons of Military Hospital and Combined Military Hospital Rawalpindi, Pakistan, after taking formal ethical clearance certificate (ERC/SA-17 dated June 30, 2017) from Army Medical College Ethical Review Committee. Sixty-eight surgeons were interviewed by non-probability convenient sampling techniques from August 2017 to March 2018. Those consultants and PG trainees were interviewed who were in scrubs and within the environment of the operation theatre and either going for or coming from surgery. After filling the consent form they were interviewed by DASS 21^6^ questionnaire; It is a WHO standardized, closed-ended questionnaire containing 21 questions. There were seven questions each for depression, anxiety and stress. it has scored from 0 to 3 (0 beings did not apply to me at all, one being applied to me to some degree, two being applied to me to a considerable degree and three being applied to me very much). Surgeons having DASS score below 13 for depression, nine for anxiety and 18 for stress were considered normal and any score above these cut off values showed a surgeon to be depressed, anxious and stressed respectively. BMI was divided into two categories; below and above 25. Physical activity includes leisure-time physical activity (for example: walking, running, swimming, hiking, cycling, playing games, sports or planned exercise) for more than or equal to 75 minutes a week. Comorbidities were present in those having diabetes, hypertension, thyroid problem, asthma and ischemic heart disease. The income per month was divided into two categories; more than 50,000 PKR or less than 50,000 PKR. After data collection, it was analysed by SPSS version 22. Frequencies and percentages were calculated for descriptive statistics. Chi-square test was applied where appropriate. A p-value of < 0.05 was considered statistically significant.

## RESULTS

The mean age of the participants was 37.4±10 years. Of total 68 surgeons participated in study 54(80%) were male and 14(20%) were female, All of the consultants were male while 23(62%) PG trainees were male. A statistically significant association was observed between consultants and PG trainees in terms of gender (p= <0.001). All except 1(3%) consultants while 18(49%) PG trainees were unmarried showing a statistically significant association between the two concerning marital status (p= <0.001). There were 10(32%) consultants and 18(49%) PG trainees having BMI below 25 kg/m^2^ and 17(55%) and 21(57%) respectively performed physical activity. Comorbidity was present in 8(26%) consultants and 7(19%) PG trainees. There was no statistically significant association between the consultants and PG trainees concerning physical activity, BMI, and comorbidity (p= 0.874, 0.171 and 0.495 respectively). There were a total of 4(22%) consultants and 16(43%) PG trainees having income per month of less than 50,000 PKR showing a statistically significant association (p= 0.012) (Table-I). The number of surgeons from different departments is shown in Table-II.

**Table-I:** Demographic variables of the study participants (n= 68).

Variables	Consultants n(%)	PG-Trainee n(%)	Overall n(%)	p- values
Age in years	46.7±8.4	29.3±2.33	37.4±10.5	-
(mean ± S.D)				
*Gender*				
•Male	31(100)	23(62)	54(79.4)	<0.001*
•Female	-	14(38)	14(20.6)	
*Marital Status*				
•Married	30(97)	19(51)	49(72.1)	
•Unmarried	1(3)	18(49)	19(27.9)	<0.001*
*Physical Activity*				
•Present	17(55)	21(57)	38(55.9)	
•Absent	14(45)	16(43)	30(44.1)	
*BMI (kg/m^2)^*				
•< 25	10(32)	18(49)	28(41.2)	0.874
•> 25	21(68)	19(51)	40(58.8)	
*Co-morbidity*				
•Present	8(26)	7(19)	15(22.1)
•Absent	23(74)	30(81)	53(77.9)
*Income per month*	24(78)	21(57)	45(66)	0.495
•50,000 PKR	4(22)	16(43)	20(44)	0.012*
•< 50,000 PKR				

* Denotes statistically significant association

**Table-II:** Surgeons from different departments (n= 68).

Departments	Consultants n(%)	PG-trainees n(%)	Overall n(%)
General surgery	1(3.22)	18(48.64)	19(27.9)
Orthopaedic	5(16.12)	8(21.62)	13(19.1)
Ophthalmology	3(9.67)	5(13.51)	8(11.8)
Cardiac surgery	7(22.58)	1(2.7)	8(11.8)
Neurosurgery	2(6.45)	3(8.1)	5(7.4)
Urology	3(9.67)	2(5.4)	5(7.4)
Spinal surgery	3(9.67)	-	3(4.4)
Otorhinolaryngology	3(9.67)	-	3(4.4)
Thoracic surgery	1(3.22)	-	1(1.5)
Paediatric surgery	1(3.22)	-	1(1.47)
Plastic surgery	1(3.22)	-	1(1.5)
Vascular surgery	1(3.22)	-	1(1.5)

**Fig.1:**
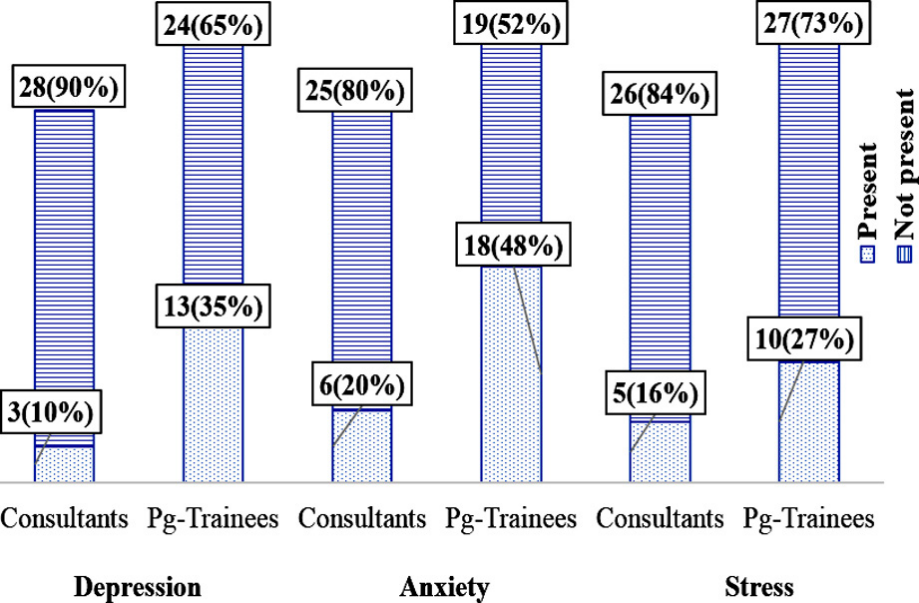
Depression, anxiety and stress among consultants and PG Trainees (n= 68).

Of total 31 consultants and 37 PG trainees, 3(10%) and 13(35%) were depressed respectively showing a statistically significant association (p= 0.014). A total of 6(20%) consultants were found to be anxious while 18(48%) PG trainees were anxious showing a statistically significant association (p= 0.012). The number of consultants stressed was 5(16%) while 10(27%) of PG trainees were found to be stressed and no statistically significant association was found between the two in terms of stress (p= 0.280). Overall results showed that 50(74%), 44(65%) and 53(78%) surgeons had no depression, anxiety and stress respectively (Fig.1).

## DISCUSSION

The results of our study were comparable to study^6^ done in India having depression, anxiety and stress levels among PG trainees as 27.71%, 36.58% and 24.24% respectively. The main reason for the high level of depression anxiety and stress in young doctors was long duty hours, not satisfied with their job, staying in a hostel and not having any hobby.

Similarly, Erdur B et al.^7^ reported that depression among surgeons was 29% which was closer to our values. Being a woman, having a low monthly income and having high depression scores were factors in their research that contributed saliently to the anxiety of doctors.

When we compared our study with Mata et al.^8^ the results were comparable, the prevalence of depression was found to be 28.3% among medical and surgical residents. The stress level in our surgeons was slightly less than that found in surgeons of New Delhi by Saini et al.^9^ which reported it to be 32.8%. In another study^10^ obesity might not directly cause depression in adolescents, as was in our study. Thus in light of these studies, surgeons being a common man may be affected in one way or another by these factors in terms of depression, anxiety and stress.

### Limitation of the study

The study was limited to one hospital understudy with a smaller sample size. Comparison of surgeons from different specialities should be included.

## CONCLUSION

In this study, the comparison of depression, anxiety and stress between consultants and PG trainee surgeons showed that there was a significant association in terms of gender, marital status and income per month. A statistically significant association showed that PG trainees were more depressed and anxious as compared to consultants.

### Author’s Contribution:

**SK:** Perceived the design and finalized the objectives along with analysing data.

**AH:** Did final drafting of the manuscript and critically analyse for important intellectual content and final approval of the manuscript and also responsible for the integrity of data.

**NK:** Did literature review, collecting and analysing data

**MK:** Did data collection, interpreting data and manuscript writing.
